# Preliminary Technical Feasibility of Integrating Auxetic Foam into Foot Orthoses for Diverse Neuropathic Etiologies: A Small-Scale Pilot Observation

**DOI:** 10.3390/bioengineering13050530

**Published:** 2026-04-30

**Authors:** LaBreesha Batey, Enrique Jackson, Changchun Zeng, Selvum Pillay

**Affiliations:** 1 Department of Mechanical and Materials Engineering, The University of Alabama at Birmingham, 1075 13th St S, Birmingham, AL 35294, USA; pillay@uab.edu; 2 Human Landing System Branch, George C. Marshall Space Flight Center, National Aeronautics and Space Administration, 4631 Saturn RD, Huntsville, AL 35812, USA; 3 Material Science Branch, George C. Marshall Space Flight Center, National Aeronautics and Space Administration, 4631 Saturn RD, Huntsville, AL 35812, USA; enrique.m.jackson@nasa.gov; 4 Department of Industrial and Manufacturing Engineering, FAMU-FSU College of Engineering, Florida Agricultural and Mechanical University, 2525 Pottsdamer St., Tallahassee, FL 32310, USA; zeng@eng.famu.fsu.edu

**Keywords:** neuropathy, ground reaction forces, gait, auxetic foams, insoles, orthotics, rehabilitation, health systems engineering, biomechanics

## Abstract

Research into auxetic foams—materials with a negative Poisson’s ratio— is expanding, yet their integration into orthotics for diverse neuropathic conditions remains largely unexplored. This pilot study evaluates the feasibility of fabricating custom auxetic foam insoles and characterizing vertical ground reaction force (vGRF) trends across a heterogeneous cohort. In collaboration with the NASA/Marshall Space Flight Center, six participants, including five representing varied neuropathic etiologies and one healthy control, performed randomized walking trials under three conditions: barefoot, over-the-counter (OTC) insoles, and custom auxetic prototypes. The healthy control was retained in the cohort-level analysis to preserve methodological symmetry across experimental conditions. To maintain physical rigor, vGRF data were mass-normalized (N/kg). A Friedman test (*n* = 6) evaluated global differences, supplemented by a dual-bootstrap analysis (1000 resamples) to quantify effect magnitudes (r) and numerical uncertainty. Although the Friedman test revealed no statistically significant global differences (Q = 0.333, df = 2, *p* = 0.846), a descriptively large effect size (r = 0.58) was observed for the auxetic material versus barefoot walking. However, wide 95% bootstrap confidence intervals prevent population-level inference, reinforcing the exploratory nature of these findings. Subject-specific observations showed descriptive differences in vGRF in three participants (0.17 to 1.18 N/kg), while increases in others occurred alongside confounding factors such as self-selected walking velocity. This work demonstrates the mechanical application of auxetic insole prototypes, providing a foundational rationale for future trials utilizing standardized walking velocity to isolate material performance.

## 1. Introduction

Peripheral neuropathy encompasses a diverse group of disorders affecting the nerves outside the brain and spinal cord [1,2,3,4,5], impacting over 20 million people in the United States alone [6]. This condition frequently impairs balance and coordination [7,8], often manifesting as altered ground reaction forces (GRFs) during locomotion [9]. Clinicians and researchers focus heavily on the vertical component (vGRF), as it serves as the primary force during gait and a metric for characterizing biomechanical patterns and observing material influence [10,11,12].

While current orthotic designs primarily target diabetic neuropathy—the most prevalent subtype [13,14,15,16]—this focus leaves a significant gap for patients with non-diabetic etiologies, such as those resulting from oncology treatments, trauma, or autoimmune conditions [17,18]. These underserved populations present distinct biomechanical needs that standardized, homogenous orthotic materials may not fully accommodate [17,18]. Evidence suggests that traditional foams often lack the specific structural tuning required to respond to altered loading patterns and variable walking velocities across such a diverse representative base [19,20,21].

**Figure 1:**
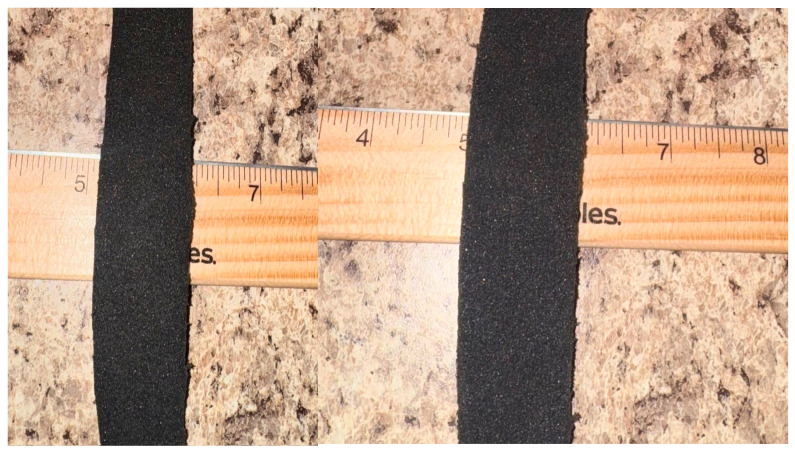

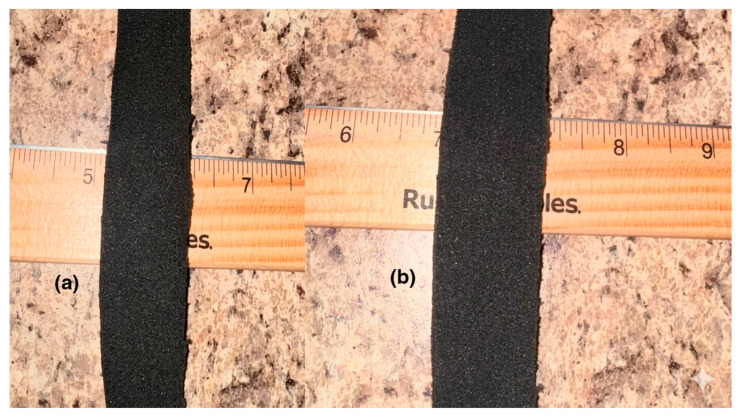
(**a**,**b**) Illustration of Negative Poisson’s Ratio in Auxetic Foam. Comparison of (**a**) the original material state and (**b**) the transverse expansion occurring during axial tension. Unlike conventional foams, the auxetic structure expands laterally when stretched, providing superior shape conformity.

To address these gaps, researchers have explored 3D-printed cellular and lattice-based designs to achieve patient-specific stiffness and plantar pressure redistribution [22,23,24,25,26]. Among these, auxetic materials—characterized by a negative Poisson’s ratio [27,28,29,30]—present a compelling mechanical advantage. Unlike traditional foams, which thin when stretched, auxetics contract laterally under axial compression and expand laterally when stretched axially [27,28,29,30] (Figure 1). This unique behavior provides superior shape conformity and energy absorption, traits already leveraged in high-performance sports footwear and protective gear [31,32,33,34]. For instance, auxetic midsoles in commercial running shoes are designed to expand in multiple directions to mimic the foot’s natural expansion during impact [34], facilitating energy dissipation and form-fitting comfort.

While the biomechanical benefits of auxetic structures are established in sports and military contexts, their application in medical orthotics for neuropathy remains largely exploratory. This preliminary study seeks to bridge this gap by evaluating the technical feasibility and mechanical application of fabricating auxetic foam insoles. Rather than providing definitive clinical proof of efficacy, this research aims to address two fundamental questions: (1) Can auxetic foams be reliably integrated into functional insole prototypes? (2) What descriptive biomechanical trends emerge when these prototypes are utilized by a diverse neuropathic cohort using mass-normalized force metrics (N/kg)? By establishing this technical foundation, this work seeks to justify larger, more rigorously controlled investigations into the mechanical influence of auxetic materials for underserved neuropathic populations.

## 2. Materials and Methods

### 2.1. Participants

A pilot cohort of six participants was recruited from the National Aeronautics and Space Administration (NASA) Marshall Space Flight Center (MSFC) (Huntsville, AL, USA). The cohort (*n* = 6) had a mean age of 54.8 ± 8.6 years (range: 43–65 years) and comprised five individuals with heterogeneous neuropathic etiologies and one healthy control (Subject E) (Table 1). Subject E was retained in all quantitative cohort analyses to preserve methodological symmetry across experimental conditions and to provide a descriptive benchmark for standard walking mechanics, rather than for direct clinical comparison. The neuropathic group included diverse clinical backgrounds to observe the material’s integration across varied conditions: diabetes (Subjects A and C), oncology-related neuropathy (Subject B), arthritis (Subject D), and injury-related neuropathy (Subject F). All participants provided informed consent as approved by the NASA Institutional Review Board (Johnson Space Center, Houston, TX, USA), and all participants in the clinical group had a confirmed medical diagnosis of neuropathy.

The extreme heterogeneity and limited size of this sample are explicitly acknowledged as primary constraints on the generalizability of the findings. Because participants were presented with distinct biomechanical baselines and etiologies, comparisons between individuals remain descriptive. Consequently, this study is framed as a case-series proof-of-concept rather than a comparative clinical trial. Furthermore, granular clinical markers—such as duration of diagnosis, quantitative sensory loss, and the presence of foot deformities—were not recorded. Given the exploratory nature of the work, a formal power analysis was not conducted; instead, the results are intended to demonstrate technical feasibility and provide observational movement data to justify future, more rigorously controlled research (further discussed in Section 4.2 regarding methodological boundaries).

### 2.2. Auxetic Foam and Insole Fabrication

The auxetic material was fabricated at the Florida Agriculture Mechanical University-Florida State University (FAMU-FSU) College of Engineering (Tallahassee, FL, USA) utilizing a patented thermo-mechanical transformation process [35,36]. Open-cell, reticulated polyurethane (PU) foam served as the precursor material. This PU foam is composed of a continuous soft phase, dispersed hard segments, and a reinforcing filler phase [36].

The manufacturing procedure comprised three sequential stages:

Mold Loading: The reticulated PU foams were placed into a mold. Dimensions were determined based on pre-calculated compression ratios required to achieve effective tri-axial compression [36].

Thermal Processing: The assembly was heated above the softening temperature of the reinforcement filler phase. This thermal energy facilitated the buckling of the foam struts into a re-entrant cellular geometry [36].

Structural Fixation: The material was cooled to room temperature while remaining under compression. Cooling below the filler’s softening point resulted in permanent deformation of the reinforcement phase, thereby locking the auxetic microstructure [36]. Properties of the auxetic foam used for insole fabrication were previously measured [37] and are summarized here.

The resulting auxetic foam was characterized by a density of 18.42 kg/m^3^, an elastic modulus of 94 ± 5 kilopascal (kPa), and a Poisson’s ratio of −0.45 [37]. This negative Poisson’s ratio allows the structure to expand laterally under tension and contract under compression [37]. For this study, foam with a thickness of 6 mm was selected for insole integration.

**Figure 2:**
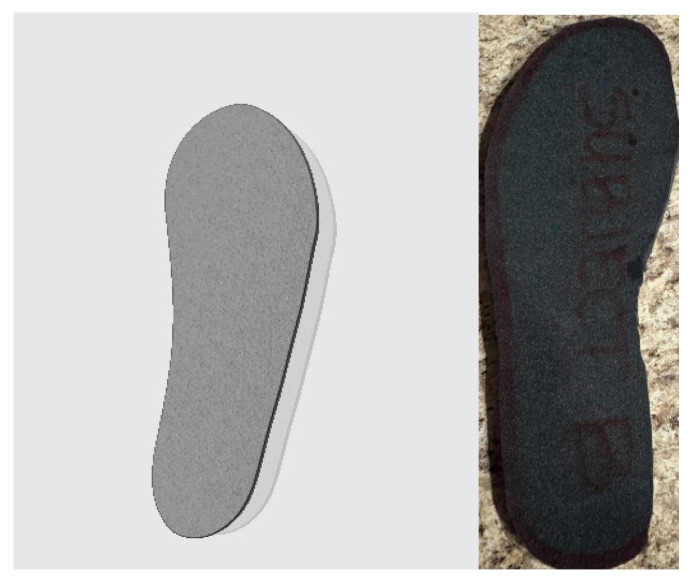
Design and Physical Prototype of the Auxetic Foam Insole. Note. (**Left**) Computer-aided design (CAD) rendering of the auxetic lattice structure. (**Right**) Photograph of the finalized foam insole prototype used during walking trials.

To ensure functional integration and proper fit, insoles were extracted from standard over-the-counter (OTC) brands —including EnduroPro (Matmarket, LLC, Portsmouth, NH, USA), Comfort Gel (Implus Footcare, LLC, Durham, NC, USA), and Athletic Works (Walmart Apollo, LLC, Bentonville, AR, USA)— to serve as templates for the pilot cohort. These templates were traced onto the auxetic foam sheets and precision cut to size (sizes 6–13) (Figure 2).

### 2.3. Procedures

**Figure 3:**
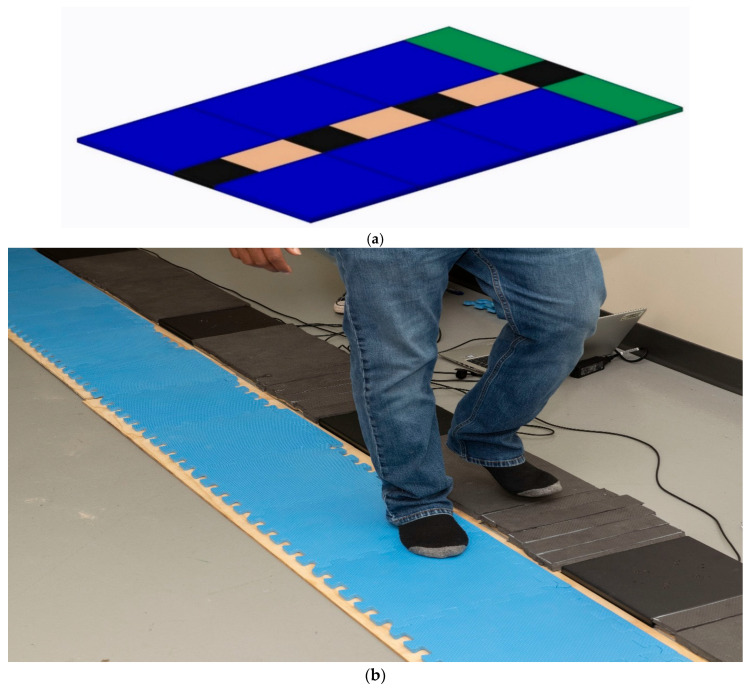
(**a**) Experimental Configuration for Gait Analysis. Schematic of the linear four-force-plate configuration used for vGRF data collection. The setup illustrates the randomized walking trials across barefoot, OTC, and auxetic footwear conditions within the test environment. (**b**) Representative Data Collection During Barefoot Walking Trials. Illustration of a participant performing a trial in the barefoot (with socks) condition. The image demonstrates the interaction between the subject’s gait and the linear force-plate array used to capture vertical ground reaction force (vGRF) profiles.

Baseline participant characteristics, including etiology and the use of mobility aids, were recorded upon arrival (Table 1). Participants performed six walking trials across a linear four-force-plate configuration (NeuLog NUL-225, S.E.S. Ltd., Rishon LeZion, Israel) (Figure 3a), with peak vGRFs recorded under three randomized conditions: (1) barefoot (with socks), (2) over-the-counter (OTC) insoles, and (3) auxetic foam prototypes. Each condition included a five-minute habituation period to allow for gait stabilization. Notably, Subject F performed additional trials both with and without clinical leg braces to evaluate the material’s interaction with high-stability orthotics; the unbraced condition was prioritized for the primary mechanical analysis.

Each trial sequence consisted of four steps, a turn, and a return to the starting position to capture bilateral data. Due to inconsistent foot strikes, data from the fourth force plate were excluded; mean peak vertical GRFs from the remaining three plates were utilized for analysis. To maintain physical rigor and reflect the acceleration-based nature of the measurement, all vGRF data were normalized by body mass and reported in N/kg. Figure 3b illustrates the representative barefoot walking condition and the corresponding GRF measurement profile.

A primary methodological constraint was the use of a self-selected walking velocity. While participants were monitored via 2D motion capture (MARVUE Camcorder, Shenzhen Guantu Technology Co., Ltd., Shenzhen, China) utilizing Windows Media Player (version 12, Microsoft Corp., Redmond, WA, USA) to identify velocity fluctuations and observe naturalistic gait, walking velocity was not externally constrained. Because vertical GRF is inherently sensitive to gait velocity [38], the observed differences in force may reflect variations in velocity rather than material influence alone. Accordingly, these data are presented as descriptive trends intended to inform future controlled trials.

### 2.4. Measures and Analysis

Mean peak vertical GRFs and qualitative comfort observations were compared across conditions. Given the exploratory nature of this feasibility study (*n* = 6), a priori power analysis was not performed and non-parametric methods were prioritized. To address the nested structure of the data and to ensure the independence of observations, peak vGRFs were averaged across trials to produce a single representative mean for each participant per condition (Table A1 and Table A2).

A Friedman test for repeated measures was used to evaluate global differences across the three footwear conditions (Barefoot, OTC, and Auxetic) at a significance level of α = 0.05. For Subject F, the unbraced condition was utilized for the primary analysis to isolate the mechanical response of the auxetic material. All vGRF values were normalized to N/kg (reflecting mass-specific acceleration) to facilitate standardized comparison across the cohort.

To characterize the magnitude and uncertainty of the observed effects, a dual-bootstrap procedure (1000 resamples) was conducted for two primary comparisons: (1) Barefoot vs. Auxetic and (2) OTC vs. Auxetic; these analyses, including the Friedman test, were performed using XLSTAT (version 2026.1, Lumivero, Denver, CO, USA).Percentile-based 95% confidence intervals (CIs) were derived to reflect the range of variability within the resampling distribution. Effect sizes (r) were calculated to provide a standardized measure of magnitude, with values of 0.1, 0.3, and 0.5 representing small, medium, and descriptively large effects, respectively.

Qualitative assessments of participant experience were collected via structured interviews (Table 1). As validated scoring systems (VAS) were not employed, these subjective results are treated as anecdotal insights. They are intended to inform future user-centered design rather than serve as primary evidence of biomechanical change. This hybrid approach prioritizes the identification of exploratory trends while explicitly acknowledging the high biomechanical variability and uncertainty inherent in this pilot cohort.

## 3. Results

In this preliminary investigation, subject-specific mean vertical GRFs (N) were evaluated across neuropathic subtypes and insole conditions (Table A1). Given the small, heterogeneous sample size (*n* = 6), these results represent individual biomechanical observations rather than generalized clinical trends. To ensure physical rigor and reflect the acceleration-based nature of the measurement, all vGRF data were normalized to N/kg (m/s^2^) (Table 2).

### 3.1. Statistical Analysis

To account for the nested data structure, a Friedman test was performed on trial-averaged means. As specified in the methodology, this analysis included the unbraced observation for Subject F to maintain a consistent sample size of *n* = 6. The global test revealed no statistically significant difference in vGRF across the three conditions (Q = 0.333, df = 2, *p* = 0.846; Table 3), suggesting the absence of a uniform directional effect across the heterogeneous cohort.

Secondary bootstrap analysis (1000 resamples) identified a descriptively large effect size (r = 0.58) for the auxetic material compared to barefoot walking (Table 3). However, the wide 95% bootstrap confidence intervals prevent inference about the direction or magnitude of effects at the population level. These intervals reflect the significant biomechanical uncertainty and variability inherent in this pilot sample. Furthermore, because walking velocity was not externally constrained, variations in individual velocity may contribute to these observations (further discussed in Section 4.2 regarding methodological boundaries).

### 3.2. Observed Subject-Specific Biomechanical Trends

**Figure 4:**
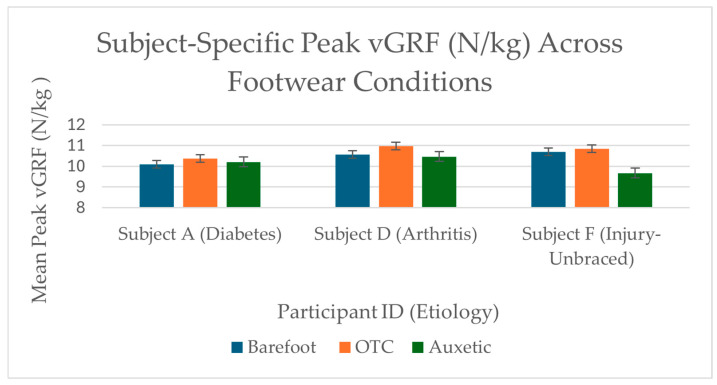
Normalized Peak vGRF Profiles for Selected Observations. Comparison of trial-averaged peak vertical ground reaction force (vGRF) normalized to N/kg (m/s^2^). For these specific participants, the auxetic prototype (green) was associated with a descriptive reduction in peak loading relative to the OTC condition. Error bars represent ±1 Standard Deviation of individual trials. These results represent individual mechanical trends; wide bootstrap confidence intervals prevent population-level inference.

Individual loading responses varied across participants. Subjects A (Diabetes), D (Arthritis), and F (Injury-unbraced) demonstrated descriptive reductions in vGRF (Figure 4), ranging from 0.16 to 1.18 N/kg relative to OTC alternatives (Table 2). The substantial reduction for Subject F when unbraced (1.18 N/kg) suggests observable changes in sagittal-plane alignment patterns when the material is not constrained by rigid external supports.

**Figure 5:**
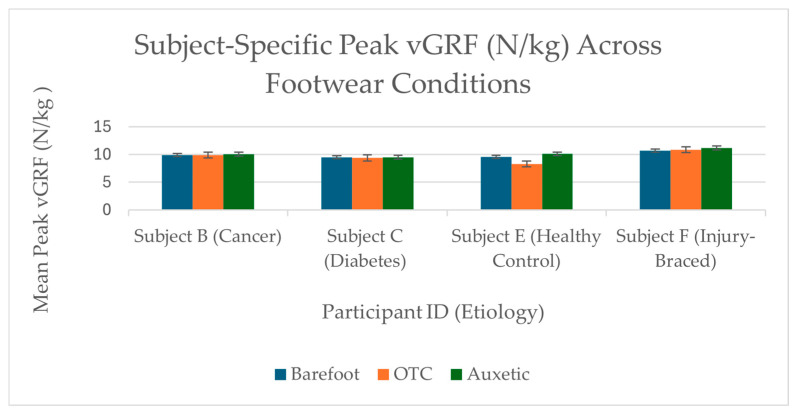
Normalized Peak vGRF Profiles for Selected Observations. Comparison of trial-averaged peak vertical ground reaction force (vGRF) normalized to N/kg (m/s^2^). In these observations, the auxetic prototype (green) was associated with a descriptive increase in peak loading. These trends occurred alongside confounding factors, including self-selected walking velocity and the mechanical rigidity of mobility aids. Error bars represent ±1 Standard Deviation of individual trials.

Conversely, increases in loading were observed in other trials (Subjects B, C, E, and F when braced) (Figure 5), most notably in the healthy control (Subject E, −1.78 N/kg). These increases occurred alongside potential confounding factors, such as self-selected walking velocity or the mechanical rigidity of mobility aids. Because walking velocity was not externally constrained, it remains difficult to isolate material performance from these individual gait variations. Given the small sample size and exploratory nature of the study, these results represent individual mechanical trends rather than generalized clinical evidence (further discussed in Section 4.2 regarding methodological boundaries).

## 4. Discussion

This feasibility study evaluated the impact of auxetic foams—multi-phase cellular materials with a negative Poisson’s ratio—on vGRF modulation. By applying systems engineering principles, this research addresses diverse neuropathic subtypes beyond diabetes, treating pathological variables and mechanical orthotic properties as inseparable components of the subject’s ambulatory system. To ensure the statistical integrity of the global analysis (*n* = 6), Subject F was represented by the unbraced condition, isolating the material’s mechanical response from the confounding influence of rigid external support.

The synthesized foam features a re-entrant structure that integrates hard domains for structural integrity with soft domains for elasticity. Unlike conventional open-cell foams, auxetics expand laterally when stretched axially and thicken under compression, providing superior shape conformity and energy absorption. While the descriptively large bootstrap effect size (r = 0.58) (Table 3) suggests the material has a measurable mechanical influence at the individual level, the wide confidence intervals emphasize that these findings are descriptive and do not permit population-level inference.

The descriptive mass-normalized reductions in Subjects A, D, and F (0.16 to 1.18 N/kg) suggest potential for load attenuation, but the simultaneous force increases in other subjects (Table 2) highlight the sensitivity of the results to confounding variables. For instance, the 1.78 N/kg increase in Subject E may be a byproduct of a naturally rapid walking velocity rather than material failure. Previous research into specialized neuropathic footwear indicates that pressure-relief effects are highly dependent on gait mechanics; standard walking patterns can increase regional loading compared to specialized antalgic or limping gaits [39]. This aligns with the observation that the increases for Subjects C and E (−0.12 and −1.78 N/kg, respectively) were potentially influenced by swifter self-selected walking velocities. For Subject B, the 0.15 N/kg increase may be attributed to the absence of standard mobility aids, whereas the contrast in Subject F—a 1.18 N/kg reduction unbraced versus a 0.30 N/kg increase braced—suggests that the mechanical rigidity of external orthotics may restrict the conforming benefits of the auxetic structure. Without standardized velocity, the extent to which the material—versus the participant’s self-selected gait—drives these peak forces remains a primary methodological boundary (see Section 4.2).

### 4.1. Qualitative Observations and Participant Feedback

**Figure 6:**
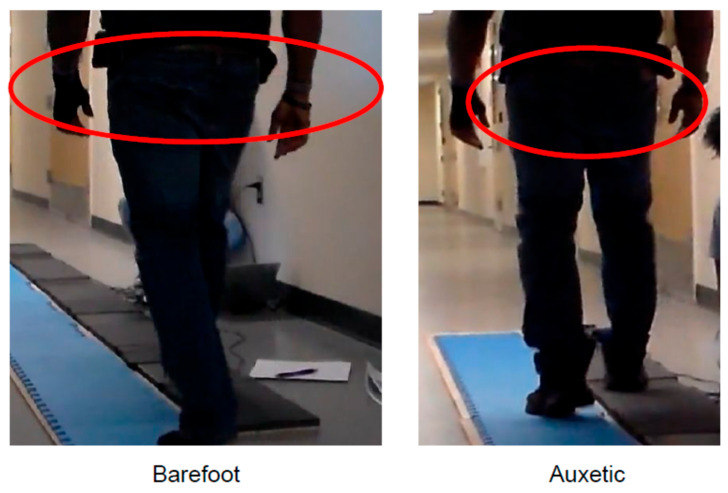
Qualitative Observation of Movement Patterns for Subject A (Diabetic Neuropathy).Motion capture stills illustrate a shift from a rotated walking pattern in the barefoot condition (**left**) to a reduction in transverse-plane foot angle when utilizing the auxetic insole (**right**); the red circles highlight the specific areas of angular change. These findings are framed as qualitative observational descriptors of movement trajectories.

**Figure 7:**
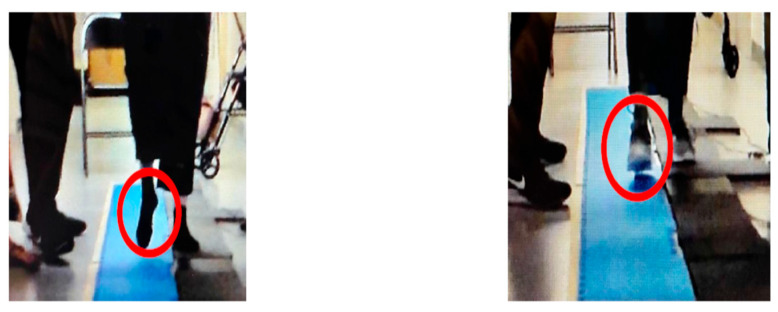
Qualitative Observations of Initial Contact Patterns in Subject D (Arthritic Neuropathy). Red circless highlight the observed difference in initial contact location, comparing the toe-first strike in the barefoot condition (**left**) to the midfoot-to-heel strike in the auxetic condition (**right**). These findings are presented as subject-specific movement observations rather than clinical evaluations of gait quality.

While the kinetic data remains exploratory, qualitative observations suggest that the auxetic prototypes influenced perceived stability and observable movement patterns. These observations provide anecdotal insight into the participants’ experience but are not presented as definitive evidence of biomechanical improvement. To maintain objective rigor, these findings are framed as qualitative observational descriptors of movement patterns rather than clinical classifications:

- **Subject A (Diabetes):** Demonstrated a shift in sagittal-plane alignment patterns, transitioning from a rotated walking trajectory to a reduction in transverse-plane rotation (Figure 6).
- **Subject B (Oncology):** Demonstrated a reduction in observed asymmetric weight-shifting. Gait pauses were observed throughout the trial, occurring concurrently with the absence of the participant’s habitual mobility aids.
- **Subject C (Diabetes):** Reported perceived changes in weight distribution; kinetic data for this observation recorded higher vGRFs alongside a swifter self-selected walking velocity.
- **Subject D (Arthritis):** Exhibited an alteration in initial contact location, characterized by a shift from a toe-first strike to a midfoot-to-heel contact pattern during the auxetic condition (Figure 7).
- **Subject E (Healthy Control):** Reported perceived stability and control; higher recorded vGRFs occurred alongside a naturally rapid walking velocity.
- **Subject F (Injury-Related Neuropathy):** Exhibited an observed alteration in sagittal-plane kinematics (e.g., decreased hip flexion during the swing phase) during the auxetic insole condition.

### 4.2. Limitations and Future Research

This study serves as an initial feasibility investigation; consequently, the results should be interpreted as exploratory. The small sample size (*n* = 6) and diverse neuropathic etiologies limit the generalizability of these findings and the statistical power of the global analysis. The wide 95% bootstrap confidence intervals (Table 3) further reflect the high biomechanical variability and inherent numerical uncertainty of a heterogeneous cohort. Additionally, the lack of granular clinical characterization—specifically regarding neuropathy duration and the severity of sensory loss—precludes the isolation of material effects from subject-specific pathology.

Future research should address these constraints by employing a larger, more homogeneous population and standardized walking velocity (e.g., utilizing a metronome or treadmill) to minimize velocity as a kinematic confounding variable. While participant feedback provided valuable anecdotal insight, subsequent studies should utilize validated scoring systems, such as the Visual Analog Scale (VAS), to quantify subjective outcomes and move beyond qualitative observational descriptors. Furthermore, the integration of advanced biomechanical metrics, including plantar pressure mapping (to observe localized loading characteristics) and formal kinematic assessment (to observe movement trajectories), is necessary to fully validate the mechanical influence of auxetic technology in neuropathic applications.

## 5. Conclusions

This feasibility investigation demonstrates the technical fabrication and mechanical application of auxetic foam insoles for neuropathic contexts. While the global Friedman test and wide bootstrap confidence intervals reflect the inherent variability of a small, heterogeneous cohort, the subject-specific observations—most notably the 1.18 N/kg difference in peak vGRF observed in Subject F (unbraced)—provide a foundational rationale for continued investigation. These findings underscore the complex interaction between material properties, representative pathology, and self-selected walking velocity.

By applying a health systems engineering framework to diverse neuropathic representative etiologies, this research establishes a preliminary baseline for the study of adaptive, shape-conforming orthotics. While the current findings are descriptive rather than definitive, this work justifies the pursuit of larger-scale, rigorously controlled trials. Future studies employing standardized walking velocity and objective kinematic assessments are necessary to further isolate material effects and characterize the mechanical influence of auxetic technology in rehabilitative applications.

## Figures and Tables

**Table 1:** Qualitative Participant Characteristics and Self-Reported Outcomes for Auxetic Insole Prototypes.

Subject	Age	Gender	Weight	Neuropathy Etiology	Shoe Size	Current Footwear and Aids	Shoe Type for Auxetic Insole	Subjective Comfort (vs. Current)	Perceived Gait Change (vs. Current)
A	43	M	205 lbs	Diabetes	10 W	Timberlands (Dr. Scholl’s)	Eduropro Comfort Gel	Positive perceived change	Reported perceived increase in gait fluidity
B	65	F	175 lbs	Oncology-related	9.5	New Balance (Memory Foam)	Athletic Works (White)	Positive perceived change	Reported perceived increase in gait fluidity
C	53	M	260 lbs	Diabetes	11	Slip-on wafers (Foam/Leather)	Athletic Works (Black)	No change observed	No change reported
D	65	F	155 lbs	Arthritis	6.5	Sketchers (No special insole)	Athletic Works (Gray)	No change observed	No change reported
E (Control)	50	F	158 lbs	N/A (Healthy)	6.5 W	Sketchers (No special insole)	Athletic Works (Gray)	Positive perceived change	No change reported
F	53	F	184 lbs	Injury-related	10	Adidas (Dr. Scholl’s) + Ankle-Foot Orthosis (AFO) Braces	Athletic Works (Gray)	Positive perceived change	Reported perceived increase in gait fluidity

*Note.* This table summarizes anecdotal participant experiences collected via structured interviews to inform future user-centered design. As validated clinical instruments (e.g., Visual Analogue Scale (VAS)) were not utilized in this pilot investigation, these results represent subjective perceptions of movement and should be interpreted as descriptive trends rather than primary evidence of biomechanical improvement.

**Table 2:** Peak vGRF Normalized to Mass-Specific Acceleration (N/kg) Across Footwear Conditions (*n* = 6).

Subject	Etiology	Weight (lb)	Mass (kg)	Barefoot (N/kg)	OTC (N/kg)	Auxetic (N/kg)	Δ (OTC-Auxetic)
A	Diabetes	205	93.0	10.09	10.37	10.21	+0.16
B	Oncology	175	79.4	9.91	9.87	10.02	−0.15
C	Diabetes	260	117.9	9.48	9.36	9.48	−0.12
D	Arthritis	155	70.3	10.57	10.97	10.47	+0.50
E	Healthy Control	158	71.7	9.55	8.31	10.09	−1.78
F	Injury (Unbraced)	184	83.5	10.70	10.85	9.67	+1.18
F	Injury (Braced)	184	83.5	10.70	10.85	11.15	−0.30

Note. vGRF values are reported in N/kg (equivalent to m/s^2^) to reflect mass-specific acceleration. Δ represents the descriptive change between standard over-the-counter (OTC) insoles and the auxetic prototype; positive Δ values indicate lower peak vGRF in the auxetic condition, while negative values indicate increased loading.

**Table 3:** Statistical Evaluation of vGRF Differences: Friedman Test and Dual-Bootstrap Analysis.

Analysis Level	Comparison	Q	df	*p*	r (Effect)	95% Bootstrap CI
Global	Barefoot/OTC/Auxetic	0.333	2	0.846	--	--
Magnitude	Barefoot vs. Auxetic	--	--	--	0.58 (L)	[−34.99, 46.55]
Magnitude	OTC vs. Auxetic	--	--	--	0.41 (M)	[−72.28, 70.17]
Magnitude	Barefoot vs. OTC	--	--	--	0.49 (M)	[−28.45, 42.11]

*Note*. Friedman and Bootstrap tests performed on trial-averaged data (*n* = 6). Effect sizes (r) calculated using a denominator of *n* = 6 to strictly reflect inter-subject response and avoid pseudoreplication. Effect magnitudes: small (0.1), medium (0.3), large (0.5). Wide CIs reflect the preliminary nature of these descriptive biomechanical observations.

## Data Availability

The data related to these studies are available upon request from the Corresponding Author.

## Abbreviations

The following abbreviations are used in this manuscript:

AFO	Ankle–Foot Orthosis
% BW	Percentage of Body Weight
CAD	Computer-Aided Design
CI	Confidence Interval
FAMU	Florida Agriculture Mechanical University
FSU	Florida State University
GRF	Ground Reaction Force
kPa	Kilopascal
NASA	National Aeronautics Space Administration
MSFC	Marshall Space Flight Center
OTC	Over the Counter
PU	Polyurethane
VAS	Validated Scoring Systems
vGRF	Vertical Ground Reaction Force

## Appendix A

**Table A1:** Subject-Specific Raw Peak Vertical Ground Reaction Forces (N) Across All Trials.

Participant ID	Condition	Mass (kg)	Trial 1	Trial 2	Trial 3	Trial 4	Trial 5	Trial 6	Mean (N)	SD
A (Diabetes)	Barefoot	93	918	938	943	953	923	956	938.50	15.48
	OTC	93	963	990	963	969	929	974	964.66	20.13
	Auxetic	93	957	961	939	925	955	958	949.16	14.15
B (Oncology-related)	Barefoot	79.4	771	804	800	793	761	793	787.00	17.10
	OTC	79.4	764	783	798	790	767	797	783.16	14.74
	Auxetic	79.4	796	826	793	784	790	783	795.33	15.85
C (Diabetes)	Barefoot	117.9	1119	1095	1165	1148	1102	1078	1117.83	33.15
	OTC	117.9	1130	1030	1111	1070	1147	1131	1103.16	44.57
	Auxetic	117.9	1106	1145	1121	1098	1127	1116	1118.83	16.51
D (Arthritis)	Barefoot	70.3	817	802	466	784	811	779	743.16	136.60
	OTC	70.3	712	798	765	792	787	775	771.50	31.49
	Auxetic	70.3	777	772	756	757	572	784	736.33	81.26
E (Healthy Control)	Barefoot	71.7	689	697	698	690	667	665	684.33	14.67
	OTC	71.7	709	703	432	388	610	730	595.33	150.02
	Auxetic	71.7	774	674	729	709	738	714	723.00	33.29
F (Injury-related)	Barefoot	83.5	912	864	896	902	892	893	893.16	16.08
	OTC	83.5	794	836	951	953	965	932	905.16	71.88
	Auxetic (Unbraced)	83.5	807	935	631	856	779	832	806.66	101.20
	Auxetic (Braced)	83.5	944	935	981	908	883	932	930.50	33.22

*Note.*Table A1 provides a consolidated record of all raw vertical ground reaction force (vGRF) trials in Newtons (N). Raw values were divided by subject mass (kg) to derive the mass-specific acceleration (N/kg) reported in the main text (Table 2). Per the methodology, the unbraced trials for Subject F were used for the primary statistical analysis (*n* = 6) to isolate the mechanical response from rigid external support. High standard deviations observed in some participants (e.g., Subjects D and E) reflect individual gait variability and the inherent challenges of force plate targeting—such as partial strikes or targeting -induced cadence adjustments—common in symptomatic populations.

**Table A2:** Subject-Specific Mass-Normalized Peak vGRF Values and Descriptive Differences (**Δ**) Across Conditions.

Subject	Etiology	Barefoot (N/kg)	OTC (N/kg)	Auxetic (N/kg)	Δ Barefoot–Auxetic	Δ OTC–Auxetic
A	Diabetes	10.09	10.37	10.21	−0.12	+0.16
B	Oncology	9.91	9.87	10.02	−0.11	−0.15
C	Diabetes	9.48	9.36	9.48	0.00	−0.12
D	Arthritis	10.57	10.97	10.47	+0.10	+0.50
E	Healthy Control	9.55	8.31	10.09	−0.54	−1.78
F	Injury (Unbraced)	10.70	10.85	9.67	+1.03	+1.18
F	Injury (Braced)	10.70	10.85	11.15	−0.45	−0.30

*Note*. Table A2 presents individual mean values and calculated differences (Δ) normalized to N/kg (m/s^2^). Positive Δ values indicate lower peak vGRF in the auxetic condition, while negative values indicate increased loading. For Subject F, both braced and unbraced iterations are provided to illustrate the mechanical interaction between the auxetic material and external supports. These mass-normalized differences served as the basis for the magnitude of effect (r) calculations in the primary analysis.
